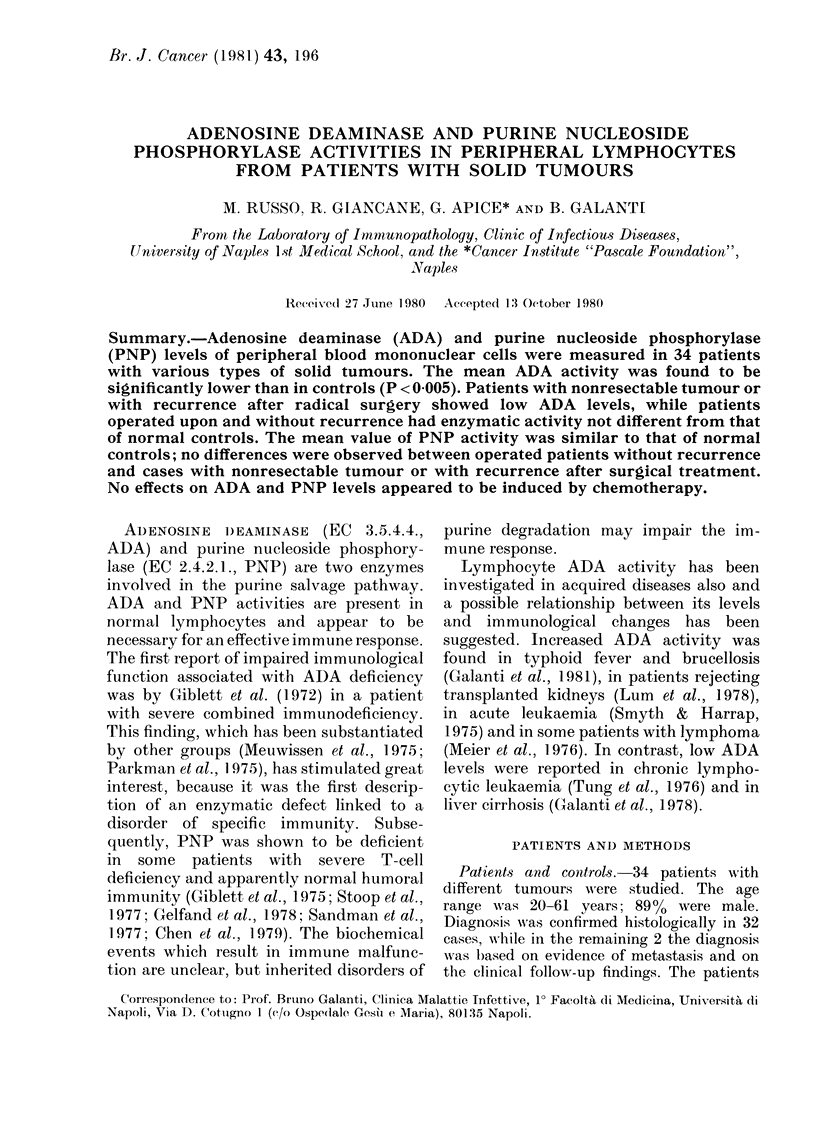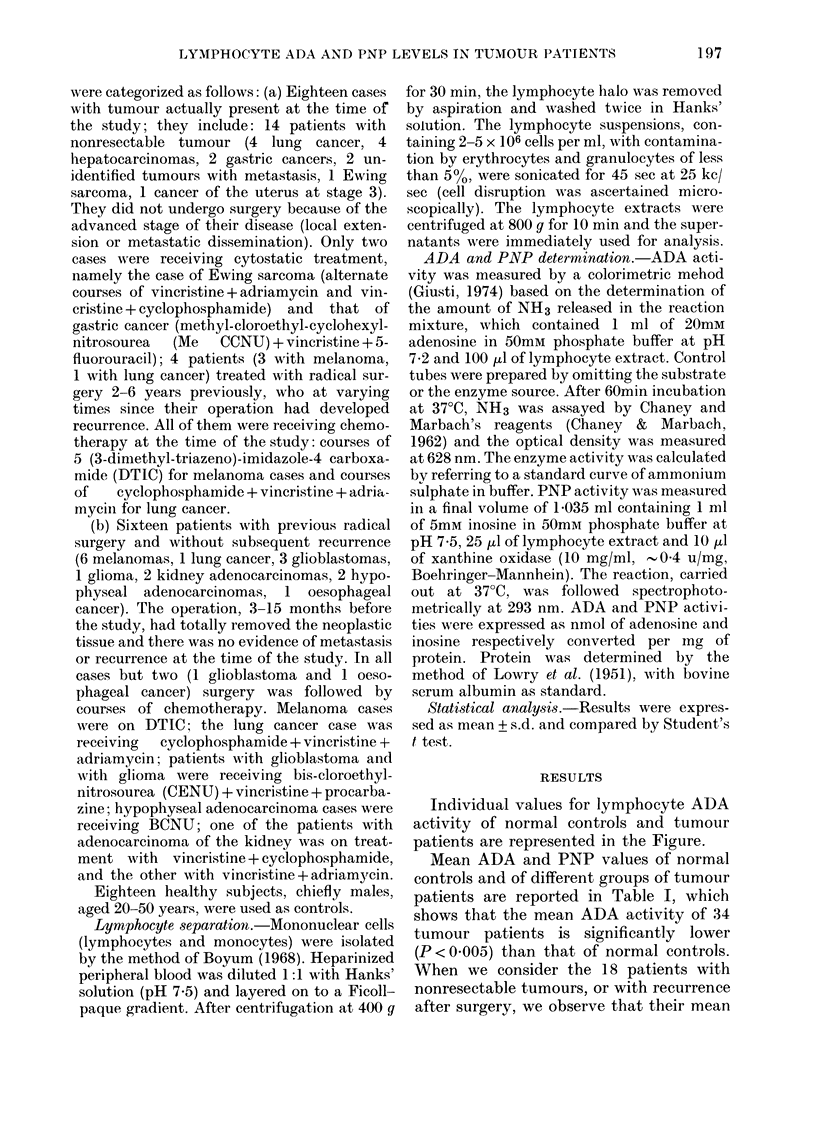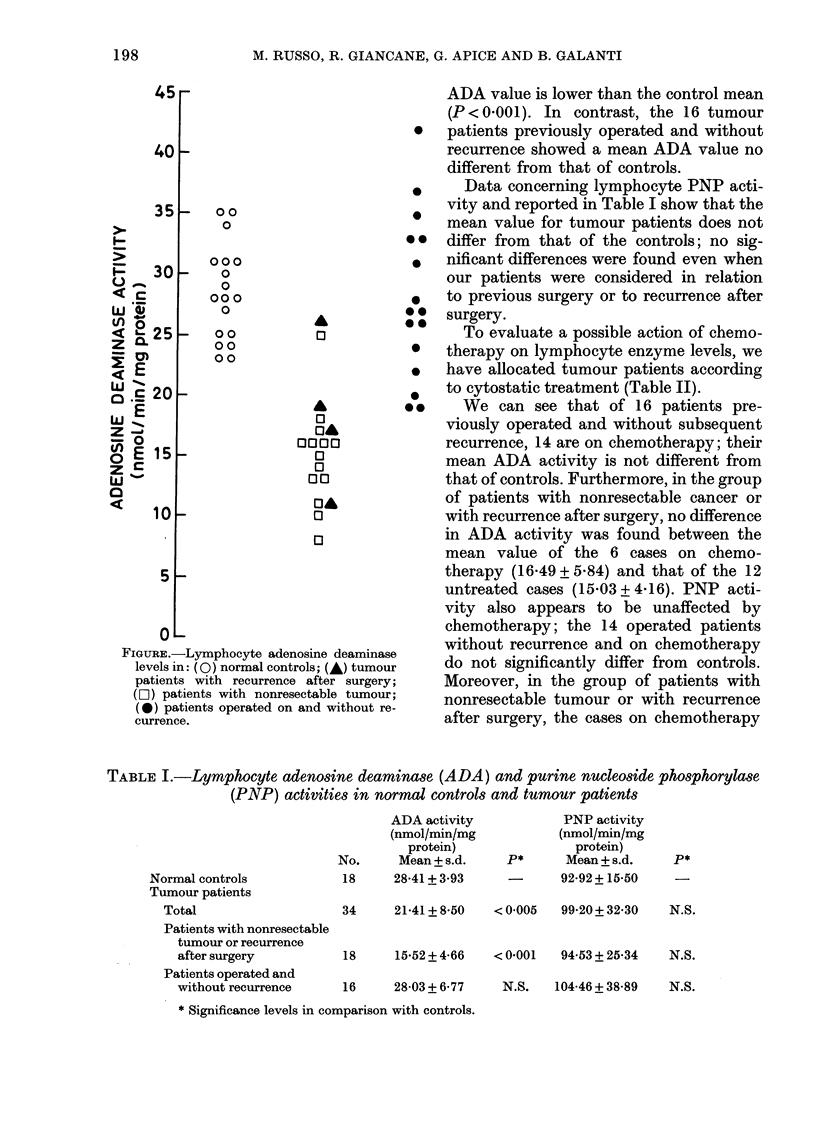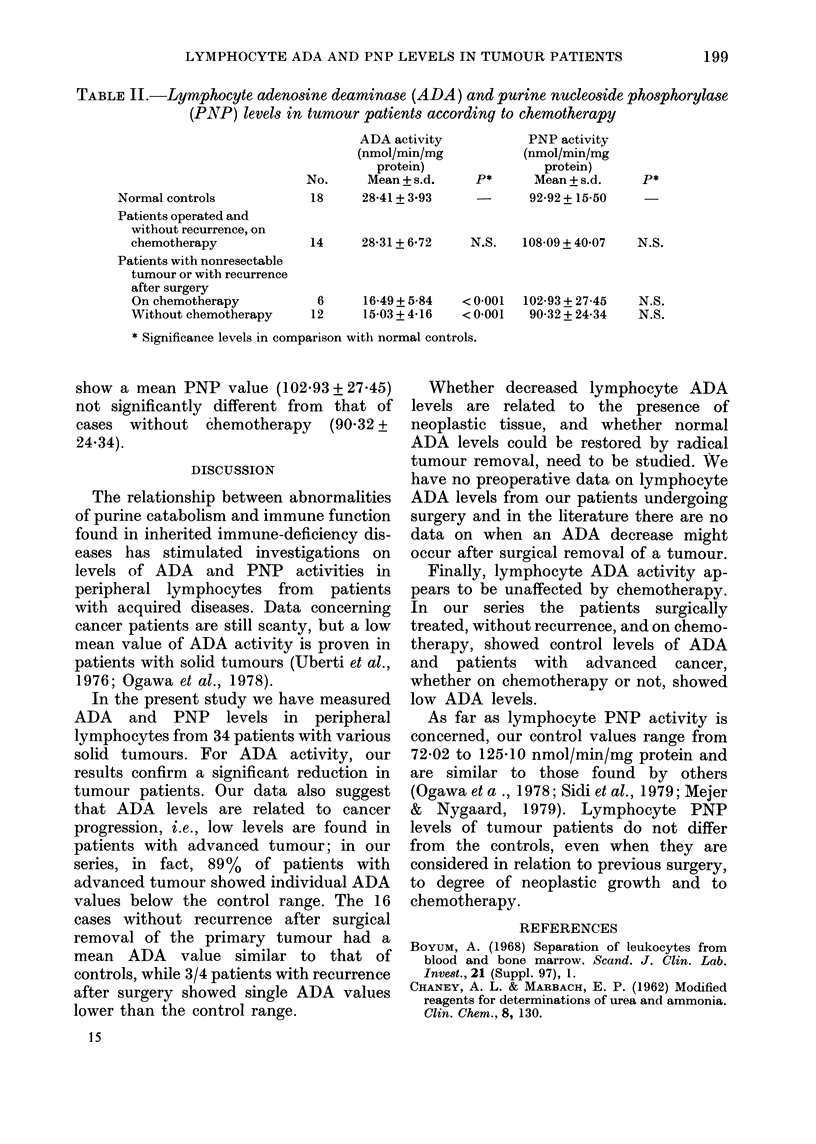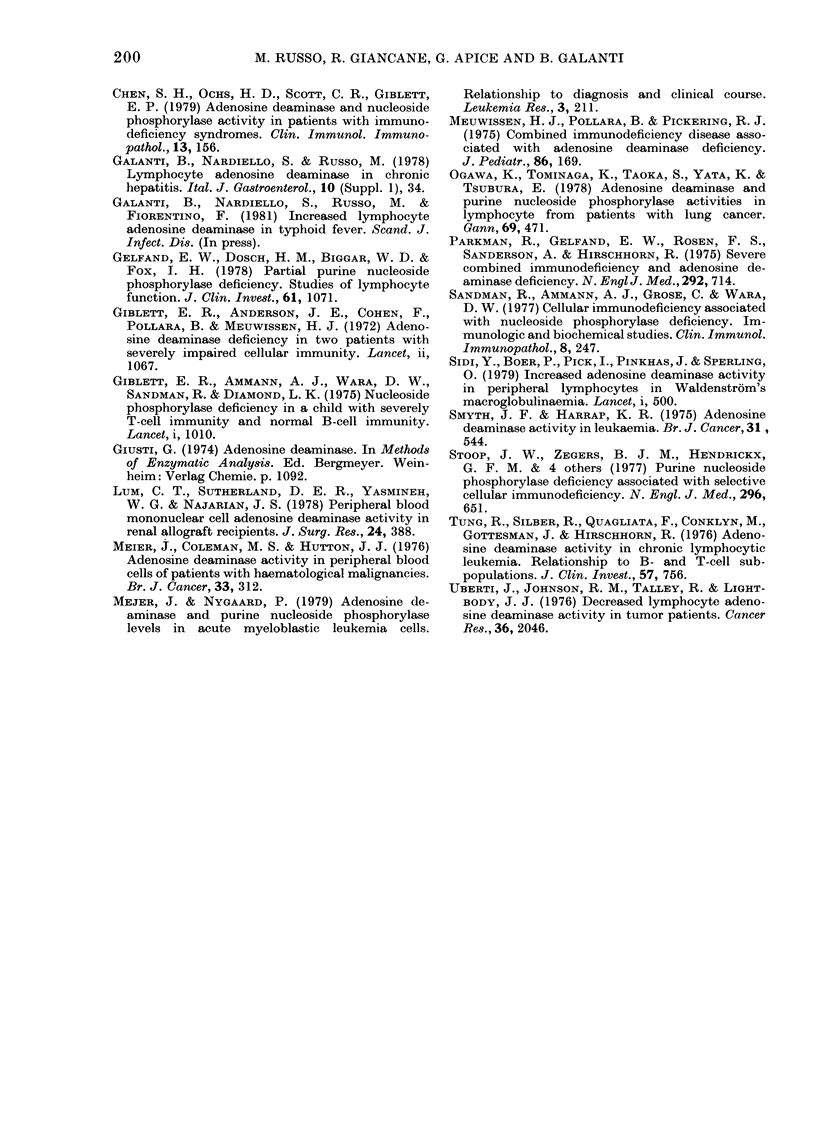# Adenosine deaminase and purine nucleoside phosphorylase activities in peripheral lymphocytes from patients with solid tumours.

**DOI:** 10.1038/bjc.1981.28

**Published:** 1981-02

**Authors:** M. Russo, R. Giancane, G. Apice, B. Galanti

## Abstract

Adenosine deaminase (ADA) and purine nucleoside phosphorylase (PNP) levels of peripheral blood mononuclear cells were measured in 34 patients with various types of solid tumours. The mean ADA activity was found to be significantly lower than in controls (P less than 0.005). Patients with nonresectable tumour or with recurrence after radical surgery showed low ADA levels, while patients operated upon and without recurrence had enzymatic activity not different from that of normal controls. The mean value of PNP activity was similar to that of normal controls; no differences were observed between operated patients without recurrence and cases with nonresectable tumour or with recurrence after surgical treatment. No effects on ADA and PNP levels appeared to be induced by chemotherapy.


*Br. J. Cancer* (1981) **43**, 196

### ADENOSINE DEAMINASE AND PURINE NUCLEOSIDE PHOSPHORYLASE ACTIVITIES IN PERIPHERAL LYMPHOCYTES FROM PATIENTS WITH SOLID TUMOURS

M. RUSSO, R. GIANCANE, G. APICE* AND B. GALANTI

*From the Laboratory of Immunopathology, Clinic of Infectious Diseases, University of Naples 1st Medical School, and the *Cancer Institute "Pascale Foundation", Naples*

Received 27 June 1980 Accepted 13 October 1980

**Summary.—Adenosine deaminase (ADA) and purine nucleoside phosphorylase (PNP) levels of peripheral blood mononuclear cells were measured in 34 patients with various types of solid tumours. The mean ADA activity was found to be significantly lower than in controls ($P<0{\cdot}005$). Patients with nonresectable tumour or with recurrence after radical surgery showed low ADA levels, while patients operated upon and without recurrence had enzymatic activity not different from that of normal controls. The mean value of PNP activity was similar to that of normal controls; no differences were observed between operated patients without recurrence and cases with nonresectable tumour or with recurrence after surgical treatment. No effects on ADA and PNP levels appeared to be induced by chemotherapy.**

ADENOSINE DEAMINASE (EC 3.5.4.4., ADA) and purine nucleoside phosphorylase (EC 2.4.2.1., PNP) are two enzymes involved in the purine salvage pathway. ADA and PNP activities are present in normal lymphocytes and appear to be necessary for an effective immune response. The first report of impaired immunological function associated with ADA deficiency was by Giblett *et al.* (1972) in a patient with severe combined immunodeficiency. This finding, which has been substantiated by other groups (Meuwissen *et al.*, 1975; Parkman *et al.*, 1975), has stimulated great interest, because it was the first description of an enzymatic defect linked to a disorder of specific immunity. Subsequently, PNP was shown to be deficient in some patients with severe T-cell deficiency and apparently normal humoral immunity (Giblett *et al.*, 1975; Stoop *et al.*, 1977; Gelfand *et al.*, 1978; Sandman *et al.*, 1977; Chen *et al.*, 1979). The biochemical events which result in immune malfunction are unclear, but inherited disorders of purine degradation may impair the immune response.

Lymphocyte ADA activity has been investigated in acquired diseases also and a possible relationship between its levels and immunological changes has been suggested. Increased ADA activity was found in typhoid fever and brucellosis (Galanti *et al.*, 1981), in patients rejecting transplanted kidneys (Lum *et al.*, 1978), in acute leukaemia (Smyth & Harrap, 1975) and in some patients with lymphoma (Meier *et al.*, 1976). In contrast, low ADA levels were reported in chronic lymphocytic leukaemia (Tung *et al.*, 1976) and in liver cirrhosis (Galanti *et al.*, 1978).

### PATIENTS AND METHODS

*Patients and controls.*—34 patients with different tumours were studied. The age range was 20–61 years; 89% were male. Diagnosis was confirmed histologically in 32 cases, while in the remaining 2 the diagnosis was based on evidence of metastasis and on the clinical follow-up findings. The patients

Correspondence to: Prof. Bruno Galanti, Clinica Malattie Infettive, 1° Facoltà di Medicina, Università di Napoli, Via D. Cotugno 1 (c/o Ospedale Gesù e Maria), 80135 Napoli.

were categorized as follows: (a) Eighteen cases with tumour actually present at the time of the study; they include: 14 patients with nonresectable tumour (4 lung cancer, 4 hepatocarcinomas, 2 gastric cancers, 2 unidentified tumours with metastasis, 1 Ewing sarcoma, 1 cancer of the uterus at stage 3). They did not undergo surgery because of the advanced stage of their disease (local extension or metastatic dissemination). Only two cases were receiving cytostatic treatment, namely the case of Ewing sarcoma (alternate courses of vincristine + adriamycin and vincristine + cyclophosphamide) and that of gastric cancer (methyl-cloroethyl-cyclohexyl-nitrosourea (Me CCNU) + vincristine + 5-fluorouracil); 4 patients (3 with melanoma, 1 with lung cancer) treated with radical surgery 2–6 years previously, who at varying times since their operation had developed recurrence. All of them were receiving chemotherapy at the time of the study: courses of 5 (3-dimethyl-triazeno)-imidazole-4 carboxamide (DTIC) for melanoma cases and courses of cyclophosphamide + vincristine + adriamycin for lung cancer.

(b) Sixteen patients with previous radical surgery and without subsequent recurrence (6 melanomas, 1 lung cancer, 3 glioblastomas, 1 glioma, 2 kidney adenocarcinomas, 2 hypophyseal adenocarcinomas, 1 oesophageal cancer). The operation, 3–15 months before the study, had totally removed the neoplastic tissue and there was no evidence of metastasis or recurrence at the time of the study. In all cases but two (1 glioblastoma and 1 oesophageal cancer) surgery was followed by courses of chemotherapy. Melanoma cases were on DTIC; the lung cancer case was receiving cyclophosphamide + vincristine + adriamycin; patients with glioblastoma and with glioma were receiving bis-cloroethyl-nitrosourea (CENU) + vincristine + procarbazine; hypophyseal adenocarcinoma cases were receiving BCNU; one of the patients with adenocarcinoma of the kidney was on treatment with vincristine + cyclophosphamide, and the other with vincristine + adriamycin.

Eighteen healthy subjects, chiefly males, aged 20–50 years, were used as controls.

*Lymphocyte separation.*—Mononuclear cells (lymphocytes and monocytes) were isolated by the method of Boyum (1968). Heparinized peripheral blood was diluted 1:1 with Hanks' solution (pH 7·5) and layered on to a Ficoll-paque gradient. After centrifugation at 400 *g* for 30 min, the lymphocyte halo was removed by aspiration and washed twice in Hanks' solution. The lymphocyte suspensions, containing $2\text{–}5 \times 10^6$ cells per ml, with contamination by erythrocytes and granulocytes of less than 5%, were sonicated for 45 sec at 25 kc/sec (cell disruption was ascertained microscopically). The lymphocyte extracts were centrifuged at 800 *g* for 10 min and the supernatants were immediately used for analysis.

*ADA and PNP determination.*—ADA activity was measured by a colorimetric mehod (Giusti, 1974) based on the determination of the amount of $NH_3$ released in the reaction mixture, which contained 1 ml of 20mM adenosine in 50mM phosphate buffer at pH 7·2 and 100 μl of lymphocyte extract. Control tubes were prepared by omitting the substrate or the enzyme source. After 60min incubation at 37°C, $NH_3$ was assayed by Chaney and Marbach's reagents (Chaney & Marbach, 1962) and the optical density was measured at 628 nm. The enzyme activity was calculated by referring to a standard curve of ammonium sulphate in buffer. PNP activity was measured in a final volume of 1·035 ml containing 1 ml of 5mM inosine in 50mM phosphate buffer at pH 7·5, 25 μl of lymphocyte extract and 10 μl of xanthine oxidase (10 mg/ml, ~0·4 u/mg, Boehringer–Mannhein). The reaction, carried out at 37°C, was followed spectrophotometrically at 293 nm. ADA and PNP activities were expressed as nmol of adenosine and inosine respectively converted per mg of protein. Protein was determined by the method of Lowry *et al.* (1951), with bovine serum albumin as standard.

*Statistical analysis.*—Results were expressed as mean ± s.d. and compared by Student's *t* test.

### RESULTS

Individual values for lymphocyte ADA activity of normal controls and tumour patients are represented in the Figure.

Mean ADA and PNP values of normal controls and of different groups of tumour patients are reported in Table I, which shows that the mean ADA activity of 34 tumour patients is significantly lower ($P < 0{\cdot}005$) than that of normal controls. When we consider the 18 patients with nonresectable tumours, or with recurrence after surgery, we observe that their mean

FIGURE.—Lymphocyte adenosine deaminase levels in: (○) normal controls; (▲) tumour patients with recurrence after surgery; (□) patients with nonresectable tumour; (●) patients operated on and without recurrence.

ADA value is lower than the control mean ($P < 0{\cdot}001$). In contrast, the 16 tumour patients previously operated and without recurrence showed a mean ADA value no different from that of controls.

Data concerning lymphocyte PNP activity and reported in Table I show that the mean value for tumour patients does not differ from that of the controls; no significant differences were found even when our patients were considered in relation to previous surgery or to recurrence after surgery.

To evaluate a possible action of chemotherapy on lymphocyte enzyme levels, we have allocated tumour patients according to cytostatic treatment (Table II).

We can see that of 16 patients previously operated and without subsequent recurrence, 14 are on chemotherapy; their mean ADA activity is not different from that of controls. Furthermore, in the group of patients with nonresectable cancer or with recurrence after surgery, no difference in ADA activity was found between the mean value of the 6 cases on chemotherapy ($16{\cdot}49 \pm 5{\cdot}84$) and that of the 12 untreated cases ($15{\cdot}03 \pm 4{\cdot}16$). PNP activity also appears to be unaffected by chemotherapy; the 14 operated patients without recurrence and on chemotherapy do not significantly differ from controls. Moreover, in the group of patients with nonresectable tumour or with recurrence after surgery, the cases on chemotherapy

TABLE I.—*Lymphocyte adenosine deaminase (ADA) and purine nucleoside phosphorylase (PNP) activities in normal controls and tumour patients*

| | No. | ADA activity (nmol/min/mg protein) Mean ± s.d. | $P$* | PNP activity (nmol/min/mg protein) Mean ± s.d. | $P$* |
|---|---|---|---|---|---|
| Normal controls | 18 | 28·41 ± 3·93 | — | 92·92 ± 15·50 | — |
| Tumour patients | | | | | |
| Total | 34 | 21·41 ± 8·50 | < 0·005 | 99·20 ± 32·30 | N.S. |
| Patients with nonresectable tumour or recurrence after surgery | 18 | 15·52 ± 4·66 | < 0·001 | 94·53 ± 25·34 | N.S. |
| Patients operated and without recurrence | 16 | 28·03 ± 6·77 | N.S. | 104·46 ± 38·89 | N.S. |

* Significance levels in comparison with controls.

TABLE II.—*Lymphocyte adenosine deaminase (ADA) and purine nucleoside phosphorylase (PNP) levels in tumour patients according to chemotherapy*

| | No. | ADA activity (nmol/min/mg protein) Mean ± s.d. | $P$* | PNP activity (nmol/min/mg protein) Mean ± s.d. | $P$* |
|---|---|---|---|---|---|
| Normal controls | 18 | 28·41 ± 3·93 | — | 92·92 ± 15·50 | — |
| Patients operated and without recurrence, on chemotherapy | 14 | 28·31 ± 6·72 | N.S. | 108·09 ± 40·07 | N.S. |
| Patients with nonresectable tumour or with recurrence after surgery | | | | | |
| On chemotherapy | 6 | 16·49 ± 5·84 | < 0·001 | 102·93 ± 27·45 | N.S. |
| Without chemotherapy | 12 | 15·03 ± 4·16 | < 0·001 | 90·32 ± 24·34 | N.S. |

* Significance levels in comparison with normal controls.

show a mean PNP value (102·93 ± 27·45) not significantly different from that of cases without chemotherapy (90·32 ± 24·34).

### DISCUSSION

The relationship between abnormalities of purine catabolism and immune function found in inherited immune-deficiency diseases has stimulated investigations on levels of ADA and PNP activities in peripheral lymphocytes from patients with acquired diseases. Data concerning cancer patients are still scanty, but a low mean value of ADA activity is proven in patients with solid tumours (Uberti *et al.*, 1976; Ogawa *et al.*, 1978).

In the present study we have measured ADA and PNP levels in peripheral lymphocytes from 34 patients with various solid tumours. For ADA activity, our results confirm a significant reduction in tumour patients. Our data also suggest that ADA levels are related to cancer progression, *i.e.*, low levels are found in patients with advanced tumour; in our series, in fact, 89% of patients with advanced tumour showed individual ADA values below the control range. The 16 cases without recurrence after surgical removal of the primary tumour had a mean ADA value similar to that of controls, while 3/4 patients with recurrence after surgery showed single ADA values lower than the control range.

Whether decreased lymphocyte ADA levels are related to the presence of neoplastic tissue, and whether normal ADA levels could be restored by radical tumour removal, need to be studied. We have no preoperative data on lymphocyte ADA levels from our patients undergoing surgery and in the literature there are no data on when an ADA decrease might occur after surgical removal of a tumour.

Finally, lymphocyte ADA activity appears to be unaffected by chemotherapy. In our series the patients surgically treated, without recurrence, and on chemotherapy, showed control levels of ADA and patients with advanced cancer, whether on chemotherapy or not, showed low ADA levels.

As far as lymphocyte PNP activity is concerned, our control values range from 72·02 to 125·10 nmol/min/mg protein and are similar to those found by others (Ogawa *et a* ., 1978; Sidi *et al.*, 1979; Mejer & Nygaard, 1979). Lymphocyte PNP levels of tumour patients do not differ from the controls, even when they are considered in relation to previous surgery, to degree of neoplastic growth and to chemotherapy.

### REFERENCES

BOYUM, A. (1968) Separation of leukocytes from blood and bone marrow. *Scand. J. Clin. Lab. Invest.*, **21** (Suppl. 97), 1.

CHANEY, A. L. & MARBACH, E. P. (1962) Modified reagents for determinations of urea and ammonia. *Clin. Chem.*, **8**, 130.

Chen, S. H., Ochs, H. D., Scott, C. R., Giblett, E. P. (1979) Adenosine deaminase and nucleoside phosphorylase activity in patients with immunodeficiency syndromes. *Clin. Immunol. Immunopathol.*, **13**, 156.

Galanti, B., Nardiello, S. & Russo, M. (1978) Lymphocyte adenosine deaminase in chronic hepatitis. *Ital. J. Gastroenterol.*, **10** (Suppl. 1), 34.

Galanti, B., Nardiello, S., Russo, M. & Fiorentino, F. (1981) Increased lymphocyte adenosine deaminase in typhoid fever. *Scand. J. Infect. Dis.* (In press).

Gelfand, E. W., Dosch, H. M., Biggar, W. D. & Fox, I. H. (1978) Partial purine nucleoside phosphorylase deficiency. Studies of lymphocyte function. *J. Clin. Invest.*, **61**, 1071.

Giblett, E. R., Anderson, J. E., Cohen, F., Pollara, B. & Meuwissen, H. J. (1972) Adenosine deaminase deficiency in two patients with severely impaired cellular immunity. *Lancet*, ii, 1067.

Giblett, E. R., Ammann, A. J., Wara, D. W., Sandman, R. & Diamond, L. K. (1975) Nucleoside phosphorylase deficiency in a child with severely T-cell immunity and normal B-cell immunity. *Lancet*, i, 1010.

Giusti, G. (1974) Adenosine deaminase. In *Methods of Enzymatic Analysis*. Ed. Bergmeyer. Weinheim: Verlag Chemie. p. 1092.

Lum, C. T., Sutherland, D. E. R., Yasmineh, W. G. & Najarian, J. S. (1978) Peripheral blood mononuclear cell adenosine deaminase activity in renal allograft recipients. *J. Surg. Res.*, **24**, 388.

Meier, J., Coleman, M. S. & Hutton, J. J. (1976) Adenosine deaminase activity in peripheral blood cells of patients with haematological malignancies. *Br. J. Cancer*, **33**, 312.

Mejer, J. & Nygaard, P. (1979) Adenosine deaminase and purine nucleoside phosphorylase levels in acute myeloblastic leukemia cells. Relationship to diagnosis and clinical course. *Leukemia Res.*, **3**, 211.

Meuwissen, H. J., Pollara, B. & Pickering, R. J. (1975) Combined immunodeficiency disease associated with adenosine deaminase deficiency. *J. Pediatr.*, **86**, 169.

Ogawa, K., Tominaga, K., Taoka, S., Yata, K. & Tsubura, E. (1978) Adenosine deaminase and purine nucleoside phosphorylase activities in lymphocyte from patients with lung cancer. *Gann*, **69**, 471.

Parkman, R., Gelfand, E. W., Rosen, F. S., Sanderson, A. & Hirschhorn, R. (1975) Severe combined immunodeficiency and adenosine deaminase deficiency. *N. Engl J. Med.*, **292**, 714.

Sandman, R., Ammann, A. J., Grose, C. & Wara, D. W. (1977) Cellular immunodeficiency associated with nucleoside phosphorylase deficiency. Immunologic and biochemical studies. *Clin. Immunol. Immunopathol.*, **8**, 247.

Sidi, Y., Boer, P., Pick, I., Pinkhas, J. & Sperling, O. (1979) Increased adenosine deaminase activity in peripheral lymphocytes in Waldenström's macroglobulinaemia. *Lancet*, i, 500.

Smyth, J. F. & Harrap, K. R. (1975) Adenosine deaminase activity in leukaemia. *Br. J. Cancer*, **31** , 544.

Stoop, J. W., Zegers, B. J. M., Hendrickx, G. F. M. & 4 others (1977) Purine nucleoside phosphorylase deficiency associated with selective cellular immunodeficiency. *N. Engl. J. Med.*, **296**, 651.

Tung, R., Silber, R., Quagliata, F., Conklyn, M., Gottesman, J. & Hirschhorn, R. (1976) Adenosine deaminase activity in chronic lymphocytic leukemia. Relationship to B- and T-cell subpopulations. *J. Clin. Invest.*, **57**, 756.

Uberti, J., Johnson, R. M., Talley, R. & Lightbody, J. J. (1976) Decreased lymphocyte adenosine deaminase activity in tumor patients. *Cancer Res.*, **36**, 2046.